# Digital Pathology and PD-L1 Testing in Non Small Cell Lung Cancer: A Workshop Record

**DOI:** 10.3390/cancers12071800

**Published:** 2020-07-05

**Authors:** Fabio Pagni, Umberto Malapelle, Claudio Doglioni, Gabriella Fontanini, Filippo Fraggetta, Paolo Graziano, Antonio Marchetti, Elena Guerini Rocco, Pasquale Pisapia, Elena V. Vigliar, Fiamma Buttitta, Marta Jaconi, Nicola Fusco, Massimo Barberis, Giancarlo Troncone

**Affiliations:** 1 Department of Medicine and Surgery, Pathology, University Milan Bicocca, 20126 Milan, Italy; fabio.pagni@unimib.it (F.P.); vdeschain@gmail.com (M.J.); 2 Department of Public Health, University of Naples Federico II, 80131 Naples, Italy; umberto.malapelle@unina.it (U.M.); pasqualepisapia89@gmail.com (P.P.); elena.vigliar@unina.it (E.V.V.); giancarlo.troncone@unina.it (G.T.); 3 Pathology Unit, San Raffaele Hospital Scientific Institute, 20132 Milano, Italy; claudio.doglioni@hsr.it; 4 Department of Surgical, Medical, Molecular Pathology and Critical Area, Pisa University, 56126 Pisa, Italy; gabriella.fontanini@unipi.it; 5 Pathology Unit, Azienda Ospedaliera per l’Emergenza Cannizzaro Hospital, 95126 Catania, Italy; filippofra@hotmail.com; 6 Pathology Unit, Fondazione IRCCS Casa Sollievo della Sofferenza, San Giovanni Rotondo, 71013 Foggia, Italy; p.graziano@operapadrepio.it; 7 Center of Predictive Molecular Medicine, University-Foundation, 66100 Chieti, Italy; antonio.marchetti@unich.it (A.M.); fiamma.buttitta@unich.it (F.B.); 8 Division of Pathology, IEO European Institute of Oncology IRCCS, 20141 Milan, Italy; Elena.Guerini@unimi.it (E.G.R.); nicola.fusco@unimi.it (N.F.); 9 Department of Oncology and Hemato-Oncology, University of Milan, 20122 Milan, Italy

**Keywords:** PD-L1, immunotherapy, NSCLC

## Abstract

A meeting among expert pathologists was held in 2019 in Rome to verify the results of the previous harmonization efforts on the PD-L1 immunohistochemical testing by scoring a representative series of non-small cell lung cancer (NSCLC) digital slides. The current paper shows the results of this digital experimental meeting and the expertise achieved by the community of Italian pathologists. PD-L1 protein expression was determined using tumor proportion score (TPS), i.e., the percentage of viable tumor cells showing partial or complete membrane staining at any intensity. The gold standard was defined as the final PD-L1 score formulated by a panel of seven lung committed pathologists. PD-L1 status was clustered in three categories, namely negative (TPS < 1), low (TPS 1–49%), and high (TPS ≥ 50%). In 23 cases (71.9%) PD-L1 staining was performed using the companion diagnostic 22C3 pharmDx kit on Dako Autostainer, while in nine (28.1%) cases it was performed using the SP263 Ventana kit on BenchMark platform. A complete PD-L1 scoring agreement between the panel of experts and the participants was reached in 57.1% of cases, whereas a minor disagreement in 16.1% of cases was recorded. Italian pathologists performed best in strong positive cases (i.e., tumor proportion score TPS > 50%), whereas only 10.8% of disagreement with the gold standard was observed, and 55.6% regarded a single challenging case. The worst performance was achieved in the negative cases, with 32.0% disagreement. A significant difference resulted from the analysis of the data separated by the different clones used: 22.3% and 38.1% disagreement (*p* = 0.01) was found in the group of cases analyzed by 22C3 and SP263 antibody clones, respectively. In conclusion, this workshop record proposed the application of a digital pathology platform to share controversial cases in educational meetings as an alternative possibility for improving the interpretation and reporting of specific histological tools. Due to the crucial role of PD-L1 TPS for the selection of patients for immunotherapy, the identification of unconventional approaches as virtual slides to focus experiences and give more detailed practical verifications of the standard quality reached may be a considerable option.

## 1. Introduction

During the last two decades, a significant revolution in pathology laboratories has been represented by the implementation of whole slide imaging and digital scanners [1]. Digital pathology may play a significant role as a didactic, diagnostic and research tool. In fact, digital slides are more efficient to adequately replicate the microscope experience than static images [2]. Another advantage in this setting is represented by the possibility to highlight and analyze interesting areas during didactic sessions [2]. Despite a more realistic experience can be obtained by adopting multi-headed microscopes, a limitation in this approach is related by the low number of viewing head extensions (5 to 14) [2] while digital slides may allow, by using dedicated monitor, an adequate experience to a higher number of attendants [3].

The evaluation of the diagnostic performances of a single tissue biomarker like programmed death-ligand 1 (PD-L1) seems to be a good opportunity to test in parallel the application of digital pathology as a routine tool in educational programs.

PD-L1 immunohistochemical (IHC) testing plays an essential role to select advanced stage non-small cell lung cancer (NSCLC) patients for treatment with immune system checkpoint inhibitors [4,5,6]. In this setting, standardization of both pre-analytical and analytical procedures is a crucial, albeit not trivial, task. Several antibodies have been adopted for PD-L1 staining and usually developed on different immunostainers. The most reproducible results were obtained with the 22C3 (Dako, Carpinteria, CA, USA), 28-8 (Dako), and SP263 clones (Ventana, Tucson, AZ, USA) [7,8]. So far, a constellation of many different research papers has been published to harmonize and standardize PD-L1 IHC assays [9,10,11]. However, interpretation remains challenging, and specific expertise is required to correctly assess PD-L1 status [12,13,14,15,16]. Various clinical interests converge towards the continuous education of pathologists in improving the reproducibility of PD-L1 analysis.

The evaluation of the diagnostic performance of a single tissue biomarker such as PD-L1 in NSCLC is a paradigmatic opportunity to implement digital pathology in highly specialized educational programs. For this purpose, a working group of Italian pathologists met in 2019 in Rome to assess the reproducibility of PD-L1 scoring using e-learning solutions. In this study, we share the results of this initiative, focusing on the reliability and affordability of this “2.0 approach” in pathology clinical practice.

## 2. Materials and Methods

### 2.1. Cases Selection

A panel of seven pathologists with broad experience in lung cancer pathology (MB, CD, GF, FF, PG, AM, and GT) selected from the archives of their respective institutions representative NSCLC histologic blocks, as an e-learning material. All cases were anonymized, and the study was fully compliant with the local ethical guidelines and the declaration of Helsinki. The study group was composed of 32 NSCLC cases, including 27 (84.4%) surgical resections and 5 (15.6%) biopsies. The latter were obtained from either primary (*n* = 3; 60%) or metastatic (*n* = 2; 40%) sites. The molecular status of epidermal growth factor receptor (*EGFR*), ROS proto-oncogene 1 receptor tyrosine kinase (*ROS1*) and anaplastic lymphoma kinase (*ALK*) was retrieved from the clinical databases. The inclusion criteria were the following: adult patients (>18 years old); no previous neoadjuvant chemoradiotherapy administered; PD-L1 testing available.

### 2.2. PD-L1 Status Assessment and Tissue Slides Digitalization

For each case, representative hematoxylin and eosin (H&E) and PD-L1 stained slides were digitized using a ScanScope CS digital scanner (Aperio, Park Center Dr, Vista, CA, USA) at a 40x magnification. The original PD-L1 protein expression was determined using tumor proportion score (TPS), i.e., the percentage of viable tumor cells showing partial or complete membrane staining at any intensity. Then, PD-L1 status was clustered into three categories, namely, negative (TPS < 1%), low (TPS 1–49%), and high (TPS ≥ 50%). In 23 (71.9%) cases PD-L1 staining was performed using companion diagnostic 22C3 pharmDx kit on Dako Autostainer, while in 9 (28.1%) cases using the SP263 Ventana kit and OptiView DAB IHC detection kit on BenchMark platform, as recommended by the manufacturer. For all cases, the PD-L1 TPS was re-assessed by the panel of pathologists by using the same classification to obtain the final gold standard. To define tumor cells as positive for PD-L1 staining two different approaches were adopted. Briefly, for the 22C3 clone, a complete circumferential or partial linear membranous staining of tumor cells at any intensity was necessary to define as positive the tumor cells, whereas any membranous and/or cytoplasmic immunoreactivity in tumor cells was considered positive for the SP263 clone. Discordant cases were discussed by the panel of pathologists in order to obtain a consensus.

### 2.3. E-Learning-Based PD-L1 Analysis Harmonization among General Pathologists

All cases were shared with 37 general pathologists who participated at the meeting using e-learning platforms. The study participants were divided into seven groups (i.e., orange, red, violet, green, yellow, pink, blue); each group was supervised by one pathologist from the expert panel as a tutor. Within the working groups, each digital case was blindly scored by each pathologist individually (TESI SPA, Milan, Italy). The automatic informatics system allowed each pathologist to express his TPS for each case on a digital table within 10 min, subsequently closing the session and saving the relative data. Finally, the informatics system stopped the timing and saved the data. A final consensus on TPS was reached by collegial discussion and the second round of scores for the digital cases.

### 2.4. Statistical Analysis

All study data were digitally recorded and collected. Subsequently, inferential statistical analysis was performed using Microsoft Excel embedded tools (EGR, FB, MJ). The inter- and intra-observer reproducibility was assessed by using positive and negative percent agreement, sensitivity (or true positive rate), specificity (or true negative rate), positive and negative predictive values. Ninety-five percent of confidence intervals were computed for all measurements.

## 3. Results

In Table 1 the clinical-pathological features of the case series are summarized. From a histological point of view, and according to the 2015 WHO classification of lung cancer, our series included 22 adenocarcinomas and 10 squamous cell carcinomas. Patients’ age ranged from 41 to 86 years old. A higher number of male patients was reported (22 vs. 10).

**Figure 1:**
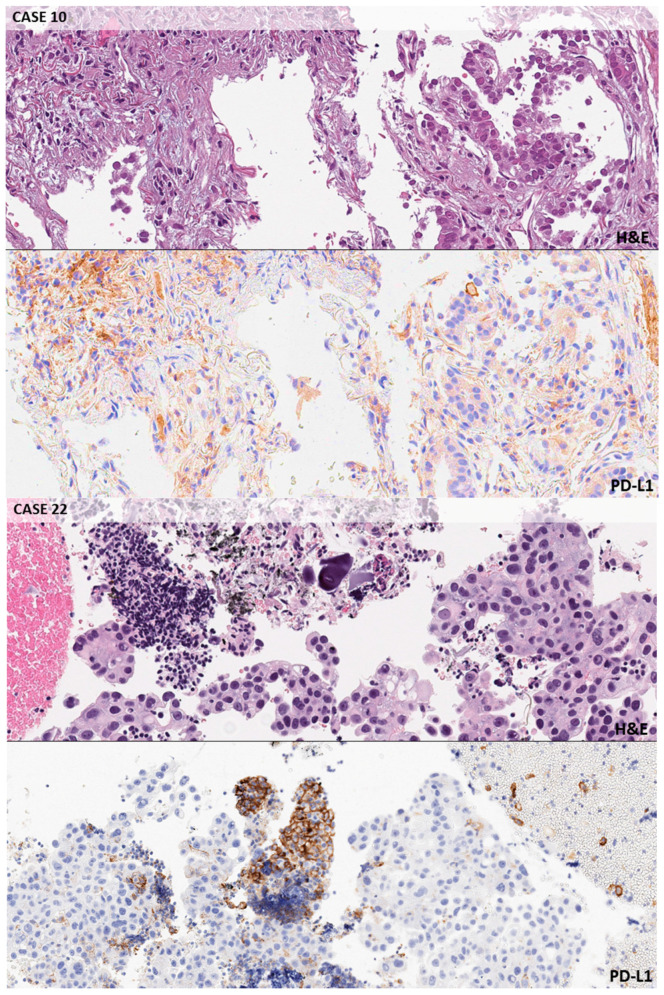
Negative cases. Exemplificative false positive background in macrophages and inflammatory cells in a lung biopsy. Case n. 10 (original magnification 10×, 22c3): PDL1 TPS < 1%; look at the background staining in macrophages peritumoral cells. CASE n. 22 (10×, 22c3): PDL1 TPS < 1%; the application of a careful magnification rule allows to classify as macrophages this group of PD-L1 strong positive cells.

**Figure 2:**
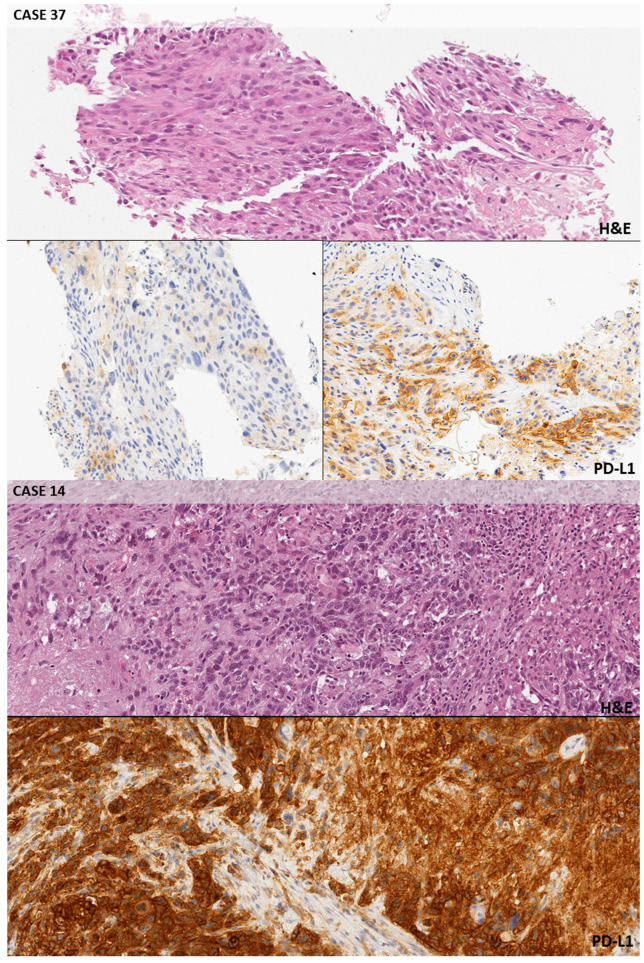

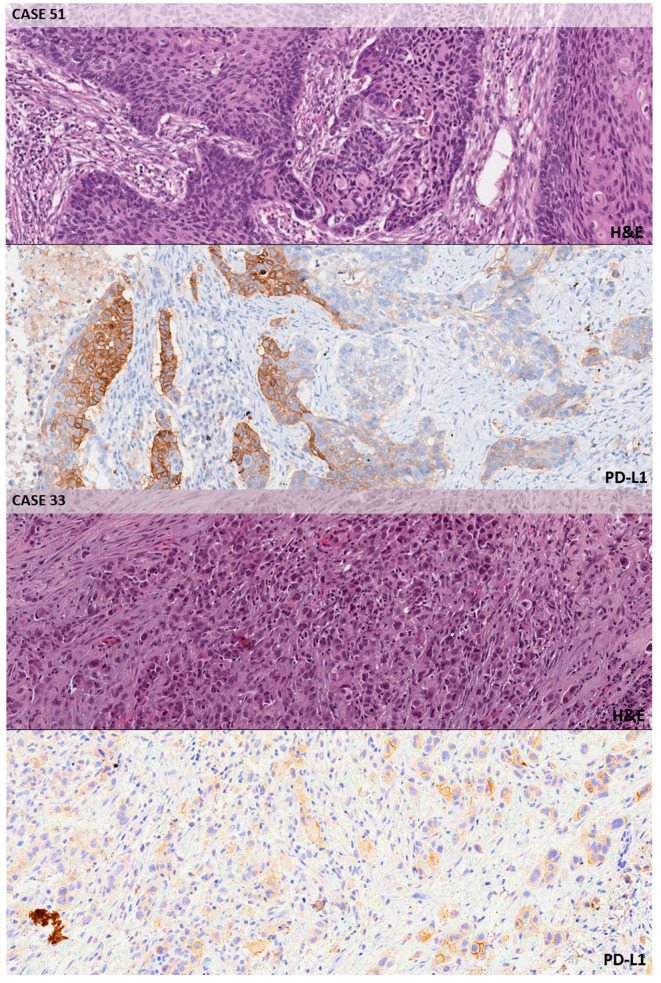
Intermediate and strong positive cases. Case n. 37 (10×, SP263): PD-L1 TPS > 50%; heterogeneous PD-L1 expression throughout the same tumor, with areas only showing faint background staining in non-neoplastic cells (bottom row, left) along with areas characterized by strong and complete membrane staining in the cancerous elements (bottom row, right). A challenging decision was jointly obtained to decide the exact threshold. Case n. 14 (10×, 22c3): PD-L1 TPS > 50%; all participants correctly ascribed this case to the ‘strong expression’ category due to the diffuse presence of PD-L1 staining in the tumor cells. Case n. 51 (10×, SP263): PD-L1 TPS 1–49%; heterogeneous PD-L1 expression with strong staining cells closely intermixed with faintly staining or negative cells. Case n. 33 (10×, 22C3): PD-L1 TPS 1–49%; a few of the neoplastic elements from this poorly differentiated adenocarcinoma showed a moderate membrane staining with the antibody, so this case was classified as an example of ‘intermediate expression’.

In 24 out of 32 selected cases (75.0%), the PD-L1 score was concordant between the original report and the expert panel of pathologists (Supplementary Table S1). In the remaining 8 cases, an under (*n* = 4) or over-estimation (*n* = 4) of positive tumor cells was reported. Seven cases were negative for PD-L1 expression, 16 were considered to be intermediate expressors, and 9 were classified as strong positive by the expert panel (Supplementary Table S1). The 37 learner pathologists, divided into 7 groups, evaluated all the digital slides on a laptop (Supplementary Table S2). The complete agreement between the expert panel (gold standard) and the participants was reached in a range of 37.5% (group red) to 78.1% of cases (group yellow); the average value was 57.1% (Table 2). A minor disagreement (1 participant per group) was recorded in 16.1% of cases (average value). In the red, orange, and green groups, two cases had no agreement with the referee scores. In Table 3, TPS disagreements were stratified according to the clinical threshold of PD-L1 scoring. The higher agreement rate among pathologists was obtained in PD-L1 high cases, with only 10.8% (36/333 instances) of disagreements with the gold standard, mainly regarding a single challenging case (55.6%, 20/36 instances; *n* = 37). Conversely, the worst performance was achieved in PD-L1 negative cases, where 32.0% (83/259 instances) of disagreements was demonstrated. Figure 1 highlights some paradigmatic challenging pitfalls due to the staining of macrophages in PD-L1 negative tumors (cases n. 10 and n. 22). In the category of intermediate expressors, 21.3% (126/259 instances) of disagreements was detected. In Figure 2, a spectrum of the possible reactions in cases near to the threshold between the intermediate and strong expressors is shown (cases n. 33 and n. 51). The specific evaluation of the participants’ performances is shown in Supplementary Table S2. The best performance of correct interpretation was 93.8% (pathologist G2). No statistically significant difference in percentage of disagreement (≥2 pathologists) with the gold standard was observed between adenocarcinomas and squamous cell carcinomas (27.3% vs. 25.7%, Supplementary Table S3). A significant difference emerged when considering the different clones adopted (Supplementary Table S4). A lower number of discrepancies (≥2 pathologists) was highlighted in the 22C3 clone group (22.3%) than in the SP263 one (38.1%, *p* = 0.01)

## 4. Discussion

Digital pathology technologies for educational, diagnostic, and research purposes are rapidly replacing the use of static images in this era of next-generation pathology. Digitalization of histologic or cytologic slides ensures the possibility to cover the entire slide surface, enabling pathologists to analyze the whole section as with the “traditional” microscope [2]. The application of e-learning during an educational pathology course may reduce the subjectivity and variability of semiquantitative analysis (by using dedicated software) and makes it possible to share interesting or challenging cases for a second opinion [17]. However, despite these possibilities several issues remain unsolved. In particular, efforts have to be devoted to validating the use of digital slides, as reported by The College of American Pathologists (CAP) and the Digital Pathology Association (DPA) guidelines [17,18]. In addition, a not negligible percentage of pathologists feel a lower compliance with digital diagnosis respect to classical microscope evaluation [17]. The gradual introduction of similar tools during informal meetings and courses may help in decreasing the reluctance of pathologists in accepting the digital slides as an option and not an enemy. The development of user-friendly platforms that contribute to the transformation of the traditional vertical didactic exposition of paradigmatic cases into more dynamic and *quiz-like* experiences is currently available especially to approach single-item issues like the evaluation of a tissue biomarker.

In the era of personalized medicine, immunotherapy with anti-PD-1/PD-L1 monoclonal antibodies represents a relevant clinical option for patients with advanced stage NSCLC [19]. In particular, taking into account the results that have been reported in KEYNOTE 024 and 042 clinical trials, the adoption of pembrolizumab should be considered as the first line or as a valid second line treatment in advanced stage NSCLC patients harboring a PD-L1 expression level ≥50% or between 1–49%, respectively [20,21]. Despite the improvements in molecular techniques, IHC still plays a key role in the evaluation of PD-L1 expression, in order to administer immunotherapy in this setting of patients. However, careful attention should be paid due to extreme intra- and inter-observer variability in the staining interpretation, in particular due to disparities in the training of pathologists [22]. In this setting, digital scanning of selected challenging cases and collegial discussion with a panel of highly trained pathologists may have an educational aim, helping pathologists to better define the expression level of PD-L1 and to ensure the best treatment choice for advanced stage NSCLC patients.

After 3 years of routine testing in the laboratories, this paper focused on the challenges in PD-L1 staining score evaluation among different pathologists by adopting digital slides. Despite a good technical confidence generally being achieved, as demonstrated in previous reports [5], several interpretative issues are remaining unsolved. To overcome these issues, a panel of seven expert pathologists and a large group of pathologists joined a meeting that raised the main diagnostic issues in specific challenging situations. All cases were re-evaluated by the panel of experts, and, after a collegial discussion, in a high percentage of cases (75.0%) an agreement with the score referred in the original report was reached.

Several pre-analytical factors may influence PD-L1 staining score evaluation. Different IHC protocols (i.e., type and duration of antigen retrieval, primary antibody dilution or incubation time, number of steps of amplification) are adopted by diverse laboratories, even when the same primary antibody is used on the same platform with the same detection kit [23,24]. Since recent literature data reported an acceptable overlapping between 22C3 PharmDx clone on Dako Autostainer or SP263 clone on BenchMark platform, the panel enrolled NSCLC cases indifferently stained by using these clones [5]. The SP263 clone demonstrates higher sensitivity than all other assays, but its specificity was lower [19]. A comprehensive meta-analysis showed the sensitivity of PD-L1 IHC 22C3 pharmDx superior than PD-L1 IHC 28-8 pharmDx and then Ventana PD-L1 (SP263) [24]. Other studies have evaluated the interchangeability of PD-L1 assays [10]. However, no data are available in prospective clinical trials. From the patient’s perspective, lower specificity may be acceptable if the progression free survival, overall survival, and adverse effects in patients with PD-L1-negative tumors treated by immunotherapy are comparable to that of conventional chemotherapy [25]. The employment of Ventana platform usually produces more intense immunoreactivity, although the morphological detail of the background tissue is not always well preserved, representing a possible issue in the distinction among tumor and the immune cells (usually alveolar macrophages). On the other hand, the Dako platform provides fairly soft immunoreactions with a better definition of the morphological features of the different cell types [26]. Although the selection of the cases enrolled in the study reflected the normal routine cases, the choice aimed to highlight the intra- and inter-tumoral heterogeneity of PD-L1 expression, which may affect the reproducibility of this analysis (Figure 2). For this reason, challenging borderline cases, characterized by a PD-L1 expression close to the proposed threshold, were included.

To better represent the routine diagnostic cases, the panel selected a mixture of small specimens (bronchoscopy and core needle biopsies), and surgical specimens of NSCLC with different histotypes and respective molecular findings (Table 1). Results were compared with the TPS assigned by the panel of experts (gold standards). The project highlighted interpretative inaccuracies potentially leading to false negative results like the suboptimal application of the magnification rule, which recommends the use of a high-power field for the scoring of mild/focal immunoreactivity [27]. On the contrary, a false positive background was a frequent source of pitfalls due to macrophages staining (Figure 1). Unspecific peri-membranous reactivity in mucous-rich adenocarcinomas may be an additional source of errors for pathologists.

An important issue was related to the evaluation of viable tumor cells. Analytical interpretation of PD-L1 staining score resulted in a false negative result if the pathologists did not establish the specimen adequacy of the tumor sample defined by at least 100 viable neoplastic cells. Another relevant aspect is the staining under-estimation when incomplete tumor membrane staining was evaluated. In previous reports, Cohen’s κ coefficient was 0.68 (95% CI, 0.65–0.71) for the 1% cut point sample set, indicating improvable inter-observer agreement in this subgroup of patients [28]. Conversely, in our series in strong positive patients a large agreement was recorded with only 10.8% of disagreements (Table 3). When considering a positive tumor cell staining around 50%, only a careful and extensive quantitative evaluation may avoid an under-estimation. Moreover, the intensity of PD-L1 staining in heterogeneous cases produced questionable interpretations (Figure 2). Literature data of a similar project showed a slightly lower 0.58 Cohen’s κ coefficient (95% CI, 0.55–0.62) for the 50% cut point sample set, indicating moderate inter-observer agreement [28]. The lower reproducibility at the 50% cut point suggested that training and external quality assessment programs should improve reproducibility of the samples with ≥50% of stained cells, particularly as this cut point is used to select NSCLC patients for anti-PD1 therapy [28]. The misclassification of patients may lead to two orders of problems: patients who do not receive the first line PD-1 blockade therapy, and patients who receive a treatment that is not beneficial [29]. All interpretation issues were a potential concern for pathologists, regardless the assay type [30]. In our study, a complete agreement between the expert panel evaluation (gold standard) and the participants was reached in 57.1% of cases (average value, Table 2). A minor additional disagreement was recorded in 16.1% of cases. The best performer featured 93.8% of correct interpretation (Supplementary Table S2).

The present paper highlights once again the potential of digital pathology. The possibility of sharing slides for educational purpose, to use WSI for agreement and quality control studies and the implementation of a digital pathology workflow for primary histological diagnosis have been widely reported [1,2,3,29]. We are approaching the third revolution in pathology with the implementation of the artificial intelligence (AI) tool even in the everyday practice with the possibility to combine the use of digital pathology and AI tool to support pathologists in the diagnostic setting [30,31]. The possibility of predicting IHC findings directly from H&E slides has also been reported [32], including the PD-L1 status by the developing of a deep learning model [33]. The obtained results were robust to interpathologist variability, opening new possibilities for PD-L1 assessment, especially when there is insufficient tissue for all the needed IHC and molecular tests.

## 5. Conclusions

In the educational setting, despite an initial reluctance, Italian pathologists’ welcome digital slides as an opportunity to share opinions and challenging cases and evaluate specific biomarkers’ performances like PD-L1. The educational digital pathology meeting planned in Rome in 2019 stressed the diagnostic challenges of the PD-L1 IHC testing, collecting NSCLC samples from the Italian national laboratories network. Despite the small number of cases (*n* = 32) and the limited inter-observer reproducibility due to the impossibility to compare the results before and after training, the projected addresses some interpretative components underlying possible sources of disagreement related to the PD-L1 testing. Due to the crucial role of PD-L1 TPS for the selection of patients for immunotherapy, the identification of educational unconventional approaches as virtual slides to focus experiences and give more detailed practical verifications of the standard quality reached may be a considerable option.

## Figures and Tables

**Table 1:** Sample set.

CASE Codes	SEX	AGE	DIAGNOSIS	*ALK*	*ROS1*	*EGFR*
1	M	76	adenocarcinoma	neg	neg	neg
2	M	76	adenocarcinoma	neg	neg	neg
3	M	78	squamous cell carcinoma	n.a.	n.a.	n.a.
4	F	76	adenocarcinoma	neg	neg	neg
5	M	78	adenocarcinoma	neg	neg	neg
6	F	54	adenocarcinoma	neg	neg	neg
7	M	65	adenocarcinoma	neg	neg	neg
8	M	67	squamous cell carcinoma	n.a.	n.a.	n.a.
9	M	56	adenocarcinoma	neg	neg	neg
10	M	68	adenocarcinoma	neg	neg	neg
11	M	61	adenocarcinoma	neg	neg	neg
12	M	74	adenocarcinoma	neg	neg	neg
14	F	62	adenocarcinoma (liver mts)	neg	neg	neg
15	M	70	adenocarcinoma (lymph node mts)	neg	neg	neg
16	M	80	squamous cell carcinoma (biopsy)	n.a.	n.a.	n.a.
17	M	64	squamous cell carcinoma (biopsy)	n.a.	n.a.	neg
20	M	86	squamous cell carcinoma	n.a.	n.a.	n.a.
22	M	75	adenocarcinoma	neg	neg	neg
30	M	68	adenocarcinoma	neg	n.a.	neg
33	M	71	squamous cell carcinoma	n.a.	n.a.	n.a.
34	F	76	adenocarcinoma	n.a.	n.a.	neg
35	F	63	adenocarcinoma	neg	n.a.	neg
36	M	55	adenocarcinoma	neg	neg	neg
37	F	74	squamous cell carcinoma	n.a.	n.a.	n.a.
38	F	71	squamous cell carcinoma	n.a.	n.a.	n.a.
41	F	41	adenocarcinoma	neg	neg	neg
45	M	71	squamous cell carcinoma	neg	neg	neg
50	F	62	adenocarcinoma	neg	neg	neg
51	M	59	squamous cell carcinoma	n.a.	n.a.	n.a.
52	M	78	adenocarcinoma	neg	neg	neg
54	F	67	adenocarcinoma	n.a.	neg	neg
55	M	72	adenocarcinoma	neg	neg	neg

Abbreviations: *ALK*: Anaplastic Lymphoma Kinase; *EGFR*: Epidermal Growth Factor Receptor; F: female; M: male; n.a.: not assessed; mts: metastasis; neg: negative; *ROS1*: ROS Proto-Oncogene 1, Receptor Tyrosine Kinase.

**Table 2:** This table reports a chromatic scale of the different diagnostic performances: green (all the participants of the group agreed with the reference score), blue (1 discordant pathologist), and red (≥2 discordant pathologists).

	*R (n = 5)*	*Y (n = 5)*	*O (n = 5)*	*V (n = 5)*	*B (n = 6)*	*P (n = 6)*	*G (n = 5)*
1							
2							
3							
4							
5							
6							
7							
8							
9							
10							
11							
12							
45							
14							
15							
16							
17							
41							
20							
22							
54							
52							
50							
37							
55							
30							
33							
34							
35							
36							
38							
51							

**Table 3:** Cases are divided according to three cut-off: PDL1 negative, intermediate and strong expressors with the aim of highlighting the most challenge cases.

**(A) PD-L1 NEGATIVE CASES (*n* = 7)**							
**Cases**	***R (n = 5)***	***Y (n = 5)***	***O (n = 5)***	***V (n = 5)***	***B (n = 6)***	***P (n = 6)***	***G (n = 5)***
3							
6							
10							
22							
30							
35							
36							
**(B) PD-L1** [1–49] expressorS **(*n* = 16)**							
	***R (n = 5)***	***Y (n = 5)***	***O (n = 5)***	***V (n = 5)***	***B (n = 6)***	***P (n = 6)***	***G (n = 5)***
1	>			<	<		<
4	<		<(3)		>(1); <(1)	<	
9		<(3)	>		<		<(3)
11	<(1); >(1)	<(4)	<(2)	<	<(4)	<(2)	<(5)
12			>				>(2)
16	>				>		>
17	<		<(3)		<(4)		<(2)
33	>						
38				<			<(2)
41						<(5)	
45							
50	>(3)	>(2)	>(2); <(1)		>(4)	>(4)	>(2)
51	>(5)	>(3)	>(3); <(1)		>(5)	>(2)	>
52	>(4)			>(5)	>(3)	>(2)	>3
54	>(4)					>(1); <(1)	
55	>						
**(C) PD-L1 STRONG POSITIVE (n = 9)**							
	***R (n = 5)***	***Y (n = 5)***	***O (n = 5)***	***V (n = 5)***	***B (n = 6)***	***P (n = 6)***	***G (n = 5)***
2							
5							
7							
8							
14							
15							
20							
34							
37							

## Supplementary Materials

The following are available online at https://www.mdpi.com/2072-6694/12/7/1800/s1, Table S1: Original PD-L1 evaluation, expert board consensus and prevalent scoring for every group; discrepancies between the original evaluation and the panel are highlighted in orange; participants errors in yellow. Table S2: Participant performances. In the columns, the cases of the study were grouped from negative (green, TPS< 1%) to strong positive (red, TPS ≥50%). In the first row, the gold standard results recorded by the board of experts were listed. In the following rows, for every pathologist who attended the meeting a progressive number and a colour of identification corresponding to the table were assigned. (ie.e R1: red table, participant number 1). Table S3: Performances by histotype. Table S4: Performances by clone.
