# Supplementary material for: Digital Pathology and PD-L1 Testing in Non Small Cell Lung Cancer: A Workshop Record
Source: Cancers (Basel). 2020 Jul 5;12(7):1800. doi: 10.3390/cancers12071800 (PMC7408471; doi:10.3390/cancers12071800)
Supplement: Supplementary file 1 [file cancers-12-01800-s001.pdf]

Supplementary Materials:

Digital Pathology and PD-L1 Testing in Non Small Cell Lung Cancer: A Workshop Record

Fabio Pagni, Umberto Malapelle, Claudio Doglioni, Gabriella Fontanini, Filippo Fraggetta, Paolo Graziano, Antonio Marchetti, Elena Guerini Rocco, Pasquale Pisapia, Elena Vigliar, Fiamma Buttitta, Marta Jaconi, Nicola Fusco, Massimo Barberis and Giancarlo Troncone

Table S1. Original PD-L1 evaluation, expert board consensus and prevalent scoring for every group; discrepancies between the original evaluation and the panel are highlighted in orange; participants errors in yellow.

CASES	ORIGINAL INSTITUTION	EXPERT BOARD	ORANGE	RED	VIOLET	GREEN	PINK	BLUE	YELLOW
1	1-49%	1-49%	1-49%	1-49%	1-49%	1-49%	1-49%	1-49%	1-49%
2	≥50%	≥50%	1-49%	≥50%	≥50%	≥50%	≥50%	≥50%	1-49%
3	<1%	<1%	<1%	<1%	<1%	<1%	<1%	1-49%	<1%
4	1-49%	1-49%	<1%	1-49%	1-49%	1-49%	1-49%	1-49%	1-49%
5	≥50%	≥50%	≥50%	≥50%	≥50%	≥50%	≥50%	≥50%	≥50%
6	<1%	<1%	<1%	1-49%	1-49%	<1%	1-49%	<1%	1-49%
7	1-49%	≥50%	≥50%	≥50%	≥50%	≥50%	≥50%	≥50%	≥50%
8	≥50%	≥50%	≥50%	≥50%	≥50%	≥50%	≥50%	≥50%	≥50%
9	1-49%	1-49%	1-49%	1-49%	1-49%	<1%	1-49%	1-49%	<1%
10	<1%	<1%	<1%	1-49%	<1%	<1%	<1%	<1%	<1%
11	1-49%	1-49%	1-49%	1-49%	1-49%	<1%	1-49%	<1%	<1%
12	1-49%	1-49%	1-49%	1-49%	1-49%	1-49%	1-49%	1-49%	1-49%
14	≥50%	≥50%	≥50%	≥50%	≥50%	≥50%	≥50%	≥50%	≥50%
15	≥50%	≥50%	≥50%	≥50%	≥50%	≥50%	≥50%	≥50%	≥50%
16	<1%	1-49%	1-49%	1-49%	1-49%	1-49%	1-49%	1-49%	1-49%
17	1-49%	1-49%	<1%	1-49%	1-49%	1-49%	1-49%	<1%	1-49%
20	≥50%	≥50%	≥50%	≥50%	≥50%	≥50%	≥50%	≥50%	≥50%
22	1-49%	<1%	1-49%	1-49%	1-49%	1-49%	1-49%	1-49%	1-49%
30	<1%	<1%	<1%	<1%	<1%	<1%	<1%	<1%	<1%
33	1-49%	1-49%	1-49%	1-49%	1-49%	1-49%	1-49%	1-49%	1-49%
34	≥50%	≥50%	≥50%	≥50%	≥50%	≥50%	≥50%	≥50%	≥50%
35	<1%	<1%	<1%	<1%	<1%	<1%	<1%	<1%	<1%
36	<1%	<1%	<1%	<1%	<1%	<1%	<1%	<1%	1-49%
37	≥50%	≥50%	1-49%	1-49%	1-49%	1-49%	≥50%	1-49%	≥50%
38	1-49%	1-49%	1-49%	1-49%	1-49%	1-49%	1-49%	1-49%	1-49%
41	<1%	1-49%	1-49%	1-49%	1-49%	1-49%	<1%	1-49%	1-49%
45	1-49%	1-49%	1-49%	1-49%	1-49%	1-49%	1-49%	1-49%	1-49%
50	≥50%	1-49%	1-49%	≥50%	1-49%	1-49%	≥50%	≥50%	1-49%
51	≥50%	1-49%	≥50%	≥50%	1-49%	1-49%	1-49%	≥50%	≥50%
52	≥50%	1-49%	1-49%	≥50%	≥50%	≥50%	1-49%	≥50%	1-49%
54	<1%	1-49%	1-49%	≥50%	1-49%	1-49%	1-49%	1-49%	1-49%
55	1-49%	1-49%	1-49%	1-49%	1-49%	1-49%	1-49%	1-49%	1-49%

Table S2. Participant performances. In the columns, the cases of the study were grouped from negative (green, TPS< 1%) to strong positive (red, TPS ≥50%). In the first row, the gold standard results recorded by the board of experts were listed. In the following rows, for every pathologist who attended the meeting a progressive number and a colour of identification corresponding to the table were assigned. (ie.e R1: red table, participant number 1).

	3	6	10	22	30	35	36	1	4	9	11	12	16	17	33	38	41	45	50	51	52	54	55	2	5	7	8	14	15	20	34	37
GOLD																																
R1																																
R2																																
R3																																
R4																																
R5																																
Y1																																
Y2																																
Y3																																
Y4																																
Y5																																
O1																																
O2																																
O3																																
04																																
05																																
V1																																
V2																																
V3																																
V4																																
V5																																
B1																																
B2																																
B3																																
B4																																
B5																																
B6																																
P1																																
P2																																
P3																																
P4																																
P5																																
P6																																
G1																																
G2																																

G 3						
G 4						
G 5						

Table S3. Performances by histotype.

	R(n=5)	*Y (n=5)*	*O (n=5)*	*V(n=5)*	*B (n=6)*	*P(n=6)*	*G(n=5)*
**Adenocarcinoma** (n = 22)							
**1**	>			<	<		<
**2**							
**4**	<		<(3)		>(1);<(1)	<	
**5**							
**6**							
**7**							
**9**		<(3)	>		<		<(3)
**10**							
**11**	<(1); >(1)	<(4)	<(2)	<	<(4)	<(2)	<(5)
**12**			>				>(2)
**14**							
**15**							
**22**							
**30**							
**34**							
**35**							
**36**							
**41**						<(5)	
**50**	>(3)	>(2)	>(2); <(1)		>(4)	>(4)	>(2)
**52**	>(4)			>(5)	>(3)	>(2)	>3
**54**	>(4)					>(1); <(1)	
**55**	>						
**Squamous cell carcinoma / NSCLC, NOS (n = 10)**							
**3**							
**8**							
**16**	>				>		>
**17**	<		<(3)		<(4)		<(2)
**20**							
**33**	>						
**37**							
**38**				<			<(2)
**45**							
**51**	>(5)	>(3)	>(3); <(1)		>(5)	>(2)	>

Table S4. Performances by clone.

	R(n=5)	*Y (n=5)*	*O (n=5)*	*V(n=5)*	*B (n=6)*	*P(n=6)*	*G(n=5)*
				**22C3 (n = 23)**			
**1**	>			<	<		<
**2**							
**3**							
**4**	<		<(3)		>(1);<(1)	<	
**5**							
**6**							
**7**							
**8**							
**9**		<(3)	>		<		<(3)
**10**							
**11**	<(1); >(1)	<(4)	<(2)	<	<(4)	<(2)	<(5)
**12**			>				>(2)
**14**							
**15**							
**16**	>				>		>
**17**	<		<(3)		<(4)		<(2)
**20**							
**22**							
**33**	>						
**34**							
**35**							
**54**	>(4)					>(1); <(1)	
**55**	>						
				**SP263 (n = 9)**			
30							
36							
**37**							
**38**				<			<(2)
**41**						<(5)	
**45**							
**50**	>(3)	>(2)	>(2); <(1)		>(4)	>(4)	>(2)
**51**	>(5)	>(3)	>(3); <(1)		>(5)	>(2)	>
**52**	>(4)			>(5)	>(3)	>(2)	>3
